# Two years of approved digital health applications in Germany – Perspectives and experiences of general practitioners with an affinity for their use 

**DOI:** 10.1080/13814788.2023.2186396

**Published:** 2023-03-15

**Authors:** Julian Wangler, Michael Jansky

**Affiliations:** Centre for General and Geriatric Medicine, University Medical Centre Mainz, Mainz, Germany

**Keywords:** Digital health applications, telemedicine, mHealth, general practitioner, primary care

## Abstract

**Background:**

Since 2020, physicians in Germany can prescribe approved digital health applications (DHAs) with the costs covered by the health system. There has so far been a lack of studies on attitudes and experiences amongst GPs in using DHAs.

**Objectives:**

The aim was to elucidate the experiences and observations of GPs that have used DHAs in health care and to examine the conditions necessary for DHAs to gain a foothold in primary care according to the GPs.

**Methods:**

In 2022, 96 qualitative semi-standardised interviews were conducted with German GPs with experience in prescribing DHAs. The GPs were all organised in digitalisation-oriented physicians’ associations. Fifty-four interviews were carried out in person and 42 by phone. The data were analysed according to qualitative content analysis.

**Results:**

Unlike health apps, the interviewees saw DHAs as reliable tools for enhancing the relationship between GPs and their patients. They saw the DHAs they had been prescribing as useful and reported various benefits, including improvements in compliance, mobility, information for patients and weight reduction. The physicians also saw room for further improvement (usability, gamification, training, information sources). Interviewees saw the inclusion of DHAs in evidence-based guidelines as a major step forward.

**Conclusion:**

The interviewees rated DHAs favourably regarding healthcare potential and as safer and more reliable than conventional health apps. Many saw benefits to healthcare from using such applications. From the interviewees’ point of view, DHAs can be integrated more effectively into patient care.

KEY MESSAGES

- GPs with experience in DHAs see overall added value in effects on healthcare, especially compared to health apps.
- Suitable conditions must prevail before DHAs can act as effective support tools for primary care.
- This requires informing GPs on the use of DHAs as well as addressing concerns.

## Introduction

In Germany, high-quality approved digital health applications (DHAs) have been integrated into standard care since 2020 by law (Improving Healthcare by Digitalisation and Innovation Act, DVG) – a step that was unique in the world at that time and still is [1]. Since then, physicians have been able to prescribe DHAs to patients with costs covered by the national health system. DHAs are set to make disease diagnostics and recognition more effective, support treatment and contribute to prevention [1–8]. Like freely available health apps, DHAs are aimed at reinforcing empowerment, motivation, and compliance as well as informing patients and encouraging a healthy lifestyle [1,3,9–11].

Coverage by the national health system is conditional upon an application being included in the national DHA directory [1,2]. This requires manufacturers to apply for approval during an audit process on various requirements (CE markings for medical devices, data protection, security standards, information quality, usability and robustness in operation, patient safety). They are also required to provide sufficient documentation for the added value of the application in its effect on healthcare [1,10]. Once these criteria have been met, the application can be included in the application directory and prescribed. A fast-track procedure is also available for preliminary inclusion in the directory if only the general criteria have initially been fulfilled [1,2,7]. This gives manufacturers one year to have the application tested pending documentation of a beneficial effect. There are various documentable categories of impact on healthcare, such as pain relief, improved information and empowerment with enhanced disease management.

GPs play a key role in successfully establishing DHAs in healthcare [12,13]. A plausible scenario would be for GPs to use such tools in targeted prevention medicine and disease management, while also managing the process of change and receiving health data from their patients regularly [1,3,4,7,12,14–19]. So far, little is known about attitudes and behavioural patterns amongst GPs concerning the use of digital health applications. The same applies to experiences in the practical use of these applications in primary care, so there is a need for a broader investigation.

### Research aims

The present study has reached an interim assessment from the viewpoint of GPs that are already interested in mHealth tools and prescribe DHAs in patient care. The focus of the study is twofold. First, it was focussed on determining these GPs’ attitudes, experiences about DHAs and their effects on healthcare. Second, it should be determined to what extent GPs perceive an additional value of DHAs compared to ordinary, freely available health apps. We aimed to deliver results as a basis to deduce the conditions necessary for tapping into the potential of digital health applications in healthcare, especially primary care.

## Methods

### Study design

Against the background of our work with the related topic of health apps [17,19,20], we regard this study as an in-depth study specifically focussed on DHAs. Consequently, there are intersections with previous studies on conventional health apps, however, there are specifics due to the described DHA approach. These were to be examined from a GP's perspective. Thus, a qualitative approach appeared most appropriate.

### Recruitment and sampling

This study involved creating a convenience sample. Thirty regional and federal physicians’ associations focussed on digitalisation were included in recruitment by post for a study that was to explore attitudes and impressions from GPs as well as their experiences of using DHAs. The associations we approached had outpatient physicians as members (in most cases mere GP associations, in some cases combined GP and specialist associations), who were engaged in discussion and regular exchanges of experience in the topic, especially in committees on quality. These physicians collaborate and keep each other up to speed in more training programmes to keep abreast of practical implementation and digitalisation and integrate digital tools in their medical practices.

We mostly approached the physicians’ associations through their websites. We contacted the medical practices acting as coordination centres for the respective association. We aimed to win only GPs from the associations mentioned. Ninety-six GPs replied, and we finally conducted the interviews with these GPs without offering incentives. We were able to run the interviews with all 90 physicians because it was possible to recruit approximately the same number of GPs in each of the 16 German federal states.

The interviews took place between March and October 2022 and were conducted by both authors (general practice researchers), each conducting half of the interviews. Fifty-four interviews were carried out in person, 42 by phone (45 to 85 min). The interviews were recorded. We sent our interviewees an explanation of the topic as well as a written declaration of consent for them to sign before the interview. The first author took care of transcription.

Table 1 provides an overview of the participating sample. All GPs included in this study are members of a physicians’ association.

**Table 1.** Sample sociodemographics (*n* = 96).

Type of practice	56 joint practices, 40 single practices
Practice setting	19 in a village or small town, 36 in a medium-sized town, 41 in a city
Status	61 practice owners, 35 GPs in employment
Age	mean: 51 years, range: 22
Gender	59 male, 37 female

### Investigation tools

Several quantitative and qualitative research studies by the authors [17,19,20] with various areas of focus on the application areas for using health apps in primary care and specialist clinical settings as well as desk research [3,5,7,10,14,16,18,21,22, inter alia] were used in designing the interview guideline (see Supplementary Appendix).

The guideline consisted of 32 open questions with four key areas derived from the authors’ preliminary studies [17,19,20]. The focal points are: Prevalence of mHealth tool use amongst patients; attitudes towards digital health applications; prescription policy and experience in using these applications in healthcare; assessment, further development, and establishment of DHAs.

### Data analysis

Both authors evaluated the transcripts using content analysis according to Mayring using the MAXQDA software [19]. This first entailed pinpointing the key statements followed by further abstraction and summarisation, finally leading to a categorised system closely based on the interview guidelines and repeatedly reviewed and modified as necessary during evaluation. Our focus lies on forming logical categories from the various opinions and experiences.

Theoretical saturation became apparent after 73 interviews. However, we had set the prior condition that all 96 interviews were to be conducted.

### Ethics

The study did not involve collecting patient data or conducting clinical tests. All 96 interviews were strictly anonymised. The Ethics Commission of the State of Rhineland-Palatinate informed us that approval by an ethics committee would not be necessary. The researchers identified the participants and requested their written consent to participate.

## Results

### Spreading of the use of mHealth

Most interviewees estimated that up to a fifth of their patients used mHealth tools, such as health apps, at least occasionally. However, users comprised a ‘*heterogeneous group*’ and ‘*you can no longer claim it was specifically younger or especially digitally minded people*’ using them (I-26f).

The lion’s share of respondents estimated the potential for patients generally interested and ready to use mHealth tools to amount to around a third, judging from their own patients at their medical practices. Most saw DHAs as ‘*a great step forwards […] in winning increasing numbers of patients over to digital forms of support in healthcare*’ (I-44m).

*We still have a long way to go in ensuring that physicians and patients understand the purpose of these apps before the opportunities of including these tools in everyday life become obvious to them. If we manage this, app usage could see a breakthrough.* (I-60m)

### Assessment of the DHA concept

Almost all participants had been introduced to DHAs early due to the digital focus of the physicians’ associations the interviewees belonged to. Some associations provided information on DHAs to the physicians straight after the new DVG law had been passed, which the interviewees described as ‘*extremely useful*’ (I-4m) as they had ‘*a form of support and consultation from the start*’ (I-28m).

This advantageous head start in information from the association’s involvement helped the vast majority of respondents recognise ‘*the clear asset that this new type of health application would be from the get-go*’ (I-74f). Around half the sample reported that despite general openness towards DHAs initially, they had ‘*not always had favourable experiences with ordinary health apps for various reasons’* in the past (I-52m).

*I was hoping for a good leap in quality from digital health applications. This refers to all those areas that ordinary health apps often fail at: Data protection and privacy, usability, reliability in collecting health data and its documentation, legal conditions, and so on. I must say that digital health applications have been a step in the right direction.* (I-56m)

DHAs are generally considered reliable tools that physicians can ‘*prescribe and recommend without uncertainty or worry*’ (I-38f). The same applied to issues regarding legal certainty, although several interviewees still had questions and expressed ‘*certain remaining doubts*’ (I-44m), especially about the risk of data collection errors and liability issues. Even so, there was still a ‘*basic trust*’ as ‘*digital health applications have a legal framework behind them placing significantly stricter demands on content quality […]*’ (I-38f).

More than half the interviewees expected DHAs to play an especially significant role or make an outstanding contribution to healthcare and/or convalescence if appropriately used; the others saw a contribution but expected this to be relatively small in what would only be a supporting role. Digital health applications rated noticeably higher than conventional health apps by importance and status in clinical healthcare. Respondents attributed this to the BfArM audit, which ‘*brings in a modicum of safety and reliability*’ (I-88f).

*Classifying these applications as medical devices was decisive. This means relatively stringent test criteria that manufacturers have to meet. But it also involves how effectively these apps can be used in disease management and prevention.* (I-56m)

### Perceived benefits and risks of DHAs

Perceived benefits from apps varied by application area. Almost all interviewees thought it would make sense if the applications helped in self-monitoring for risk factors such as weight, blood pressure, and blood sugar; lifestyle changes such as diet, quitting smoking, coping with psychological problems; as well as preventive measures and medication management. Two out of three respondents favoured direct support in monitoring and treating chronic diseases.

Respondents especially saw increased motivation and compliance as the greatest benefits of using DHAs in a clinical setting (Table 2). Increased empowerment, health literacy, and reaching new patient types were also significant.

**Table 2.** Benefits and drawbacks of digital health applications.

Question: *What are the most important benefits of using digital health applications in a clinical setting? Where would you see drawbacks or risks? (n* = 96)	Number of mentions
Benefits	
Enhancing motivation	71
Increasing empowerment	68
Improving compliance (inter alia medication adherence)	64
Improving appointment management	56
Reinforcing health awareness and education	56
Making treatment more individual and effective	49
Reaching new patient types	46
Earlier detection, diagnosis, and treatment of disease or risk of disease	44
Drawbacks	
Lack of data privacy, personal data protection	48
Measurement errors or treatment failure due to complicated use	44
Raising or inculcating health anxiety	34
Impersonal doctor-patient relationship	25

*Note:* The logical categories presented in this table were formulated by the authors as described in the Methods section under ‘Data analysis’.

*I have often seen a clear difference in disease management depending on whether a patient feels powerless and passive, or like an active participant, from working with patients. […] Digital health applications could effectively reinforce this feeling of empowerment and co-management*. (I-64m)

Some physicians also pointed out the potential effectiveness and efficiency benefits of doctor-patient networking, such as measuring health data using DHAs and transferring them to the medical practice, ideally by integrating them into the office software system. Several interviewees saw this as a possibility for treating diseases and health risks in a more targeted and individual manner.

Regarding risks, some respondents reported concerns on lack of safety and specifically data privacy such as from existing data leaks despite the BfArM audit. Some also saw undesirable effects such as measurement errors due to insufficient suitability for certain patient types – ‘*especially with complex applications that are not intuitive*’ (I-34m). This could lead to incorrect health data being collected or, in extreme cases, treatment failure. One negative consequence might be that using digital applications unwisely could cause worry among patients already suffering from health anxiety. For example, a certain type of user logic in the application could prompt misinterpretation or limit fixation on certain parameters.

*Looking at it realistically, using these digital applications presents opportunities and risks in equal amounts. This makes medical judgement and control crucial. What application could I reasonably expect which patient to use? Only a doctor can make a qualified assessment of this kind, but of course, he or she also needs the relevant background knowledge or at least an idea of where to get information as to which application could be useful for what type of patient.* (I-46f)

*Where can I get this information? What information platform would guide me to the right digital health application for the right patient?* (I-89m)

### Initial reasons for using DHAs

Respondents reported various combinations of causes on why they initially saw DHAs as exciting and worthwhile. They quoted care deficits such as compliance-sensitive treatment support and sustainable lifestyle changes as prevention. Recommendations from colleagues and curiosity about digital tools also played a role. Again, many spoke highly of the reliable information they had been provided by the physicians’ associations.

Over half the interviewees reported advising patients to use certain conventional health apps before DHAs were introduced, so there was some experience in using digital health apps, albeit haphazard.

*My previous experience with health apps comes from individual cases. These individual cases mainly resulted from patients having already used eHealth tools or showing great willingness to use them. So, I didn’t do much or initiate the process. That has changed with digital health applications.* (I-66f)

The willingness to recommend and use DHAs has changed according to many respondents. Around two-thirds reported that mHealth apps in medical practice had gained some regularity – ‘*albeit at a limited level so far*’ (I-38f). The increase in trust and reliability from the DVG law and the DHA concept provided the primary justification.

*I approach patients proactively and raise the option of support from digital health applications whenever the situation arises.* (I-30f)

### Areas of application and expectations towards DHAs

DHAs were mainly prescribed for prevention and self-control, lifestyle, and promoting exercise as specific application areas. Applications that often came up covered lifestyle changes in type 2 diabetes mellitus and severe obesity as well as prevention through exercise, dealing with depressive episodes, sleep disorders, and tinnitus.

The interviewees raised the importance of DHAs being easy to understand and use with a clear design for them to consider recommending one. DHAs should protect personal data the most effectively possible while providing customisation options and encouraging patients to become more health-conscious through gamification. A significant share of the sample emphasised that doctors needed solid and reliable sources of information on the respective application as a further requirement. Some respondents named permanent inclusion in the DHA directory as a mandatory requirement for them to prescribe it.

Almost all physicians responded that their prescribed applications had proven useful overall when asked about their general experiences. Benefits to healthcare and/or convalescence were widely observed. These especially applied to factors such as improved compliance and self-management in chronic disease, increased mobility, and noticeable weight reduction (Table 3). DHAs showed the most added value in prevention and self-control, health-oriented lifestyle and exercise promotion. Seven interviewees reported adverse effects – overcomplicated design overwhelming patients trying to use the app and negative impact on patients with health anxiety.

**Table 3.** Beneficial effects observed from using approved digital health applications.

Question: *What health effects have you seen from patients using approved digital health applications?* (*n* = 96)	Number of mentions
Increased compliance, such as in taking medications and adherence to treatment	83
Improvement in health awareness and education	65
Improvement in self-management, such as in chronic disease	64
Weight reduction such as BMI, abdominal circumference, waist circumference	59
Increased mobility	62
Stable decrease in blood sugar (HbA1c)	41
Prevention of sequelae, such as diabetic foot syndrome and CHD	40
Decrease in complications, such as hypoglycaemia	38
Regression of psychological side effects, such as depression	37
Regression of metabolic syndrome	29
Elimination of the need for more severe treatment options, such as insulin therapy	25

*Note:* The logical categories presented in this table were formulated by the authors as described in the Methods section under ‘Data analysis’.

### Further development approaches

Physicians with user experience outlined various areas of focus when responding to an open question on how to make DHAs more accessible and, therefore, more attractive for use in (general) medical care (Table 4). Many physicians lacked factual, reliable information on DHAs. There was much criticism of the DHA directory as it is not detailed enough and sometimes came too close to manufacturer information. In some cases, this was also linked to a more general criticism on what justifies the fast-track procedure. Some respondents suggested the national health portal as a viable information platform focussing on digital health applications.

**Table 4.** Approaches towards easing the integration of approved digital health applications in clinical healthcare.

Question: *Judging from your previous experience with approved digital health applications, how do you think digital health applications could be improved, and what would you like to see?* (*n* = 96)	Number of mentions
Reliable information platform from a reliable source focussing on digital health applications ideally managed by the state (one of the suggestions offered: German National Health Portal)	67
Optimisation or further optimisation of usability in digital health applications, especially towards simpler, more intuitive and target group-specific use to prevent measurement errors and incorrect use	60
Training programmes for physicians on using digital health applications, especially in primary care, with sufficient CME-certified training	49
More gamification elements, more interactive and light-hearted approach to patient guidance	45
Appropriate remuneration for medical services and additional effort involved with digital health applications on the German national medical fee schedule	45
Inclusion of digital health applications in (evidence-based) guidelines and other instructions, such as from professional organisations	42
Digital health applications should be designed to prevent health anxiety, such as by eliminating the possibility of misinterpretation by patients or focussing on individual health parameters	38
Increased advice and support in using digital health applications for patients from statutory health insurance organisations	36
Cancellation of the fast-track procedure for temporary application approval, tightening the evaluation procedure	32
Technical aspects of integrating digital health applications in medical practice, such as through cost-neutral functional connection to office software	29
Improvement and further improvement of information and data privacy and protection by drawing up more binding and uniform data protection standards for manufacturers	28
Clear exclusion of liability risks for physicians if, for example, a treatment error occurs due to a bug in a digital health application – responsibility and liability should not lie with service providers or patients	25
Members of all statutory health insurance organisations should receive bonuses or bonus programmes for using certain digital health applications regularly and transferring the data to the respective organisation	14

*Note:* The logical categories presented in this table were formulated by the authors as described in the Methods section under ‘Data analysis’.

Despite the high level of satisfaction with the DHAs used, potential was seen in improving the user experience and usability as well as extending interactivity and gamification. Respondents also saw the inclusion of DHAs in evidence-based guidelines as a major step forward. Interviewees advocated a comprehensive further training programme for physicians towards encouraging more use of these applications in primary care. Many respondents reported that their associations had provided them with extensive information on using DHAs. The problem was that many GPs in Germany have little or no knowledge of the legal framework provided by the DVG law.

*Sufficient information is crucial in the acceptance and willingness to allow digital health applications, also obviously with skills in dealing with them. […] But one thing shouldn’t be underestimated: It’ll be a tough ride if you don’t have a starting point to work from and you don’t even know what digital health applications are all about.* (I-59m)

Interviewees emphasised that DHAs should not lead to misinterpretations amongst patients or a limiting fixation on certain parameters being suggested due to the app’s user logic. Many were concerned that the widespread use of DHAs might trigger a self-medication process in certain patients, resulting in doctors’ advice declining in importance.

*How can we make sure that these applications don’t start a kind of self-therapy for patients? You know this self-medication and self-research online has been taking on a life of its own. One word – ‘Cyberchondria.’ Even if I think these applications are up-and-coming, I’ve also seen what this trend can do especially in patients with health anxiety.* (I-41f)

Many GPs wished for health insurance companies to approach patients with more advice and support in using DHAs. Even as it stands, statutory health insurance organisations will provide DHAs without an explicit doctor’s prescription, given the corresponding indication [9].

## Discussion

### Main findings

Our interviews showed that GPs with experience in health apps rated DHAs favourably regarding healthcare potential and as much safer and more reliable than conventional health apps. Such applications were especially beneficial in supporting prevention, self-control, and lifestyle changes. The same applies to practical experience with DHAs: Respondents mainly reported observing beneficial healthcare effects.

Despite the favourable general assessment of DHAs and their potential use, GPs still show limited willingness to use these applications extensively and consistently in patient care. This is mainly justified with a little overview of mHealth tools and their possible applications. The interviewees expressed a need for neutral research sources focussed on health apps.

Regardless of the potential areas of application for DHAs, interviewees reported criticism on lacking documentation of efficaciousness and liability issues, especially regarding diagnostic and therapeutic DHAs. A sizeable number of respondents saw a specific problem in the fast-track procedure.

To promote the use of DHAs in the GP setting, respondents expressed the desire to see a wide range of professional CME-certified training programmes. With regard to further optimisation, physicians with experience in DHAs emphasised reinforcing motivation-boosting usability with an intuitive user interface.

### Comparison with existing literature

Health apps can benefit diseases such as obesity and diabetes mellitus type 2 by documenting symptoms and encouraging changes in lifestyle, such as diet and exercise [3–8]. Studies have shown GPs to see potential benefits in health apps but have so far been reluctant to integrate mHealth tools into patient care due to concerns about safety issues and reliability as well as implementation in everyday clinical practice [12,17–20,23,24]. This comes along with great uncertainty in selecting suitable apps from a dynamic application market.

The DVG law aims to create a basis for implementing DHAs in healthcare using clear-cut quality standards [1,7]. So far, Germany is the only European country in which state-approved health apps can be included in the reimbursement of the health system under certain conditions. It can be assumed that other countries might use the implementation of DHAs in Germany as a basis or role model for their own decisions [1,10].

Compared to previous surveys, our interviews showed the image and acceptance of DHAs to be noticeably more pronounced than in ordinary health apps among the GPs interviewed in the present study [17,19,20]. Respondents showed greater overall confidence in DHAs as solid, relatively safe, and potentially effective applications due to the necessary examination for inclusion in the DHA directory and legal framework – a finding also hinted at in a Barmer health insurance survey [25]. This promises favourable conditions for implementation in primary care.

Studies have pointed out that a limiting factor in the willingness of GPs to use digital tools and mHealth apps in patient care is related to their assessment of competence about the application of such tools [14–16,19]. Also, the present study has shown that this self-assessment can be demonstrated based on two aspects. First, lack of experience with mHealth programs means that most respondents require confidence in their ability to guide and support patients in using DHAs [18]. Second, reliable information sources are needed [14]. Cementing DHAs in general practice across the board would require informing GPs on the fundamentals of the law (DVG), while addressing concerns and requests.

Several studies have shown that GPs are dissatisfied with the transparency and reliability of information sources currently available to support patients with prescription or freely available health apps [12,16,17,19,20,25]. Not only in this study, German GPs suggested the national health portal as a possible information platform with this specific focus [21,23]. Professional organisations and their official publications could provide support with their own information services and discussion on healthcare outcomes from using DHAs. Also an authority is needed to provide an overview of which applications are suitable for which area and what needs consideration when using them. The present study found that physicians’ associations could play a key role in providing and sharing information on mHealth topics.

A discussion subject is the fast-track procedure for approving new DHAs criticised by the GPs surveyed in this study. This procedure allows provisional inclusion of apps in the directory without evidence of beneficial healthcare effects. An assessment on healthcare system digitalisation from the German Council of Experts highlighted the essential nature of careful evaluation of an app’s effectiveness and benefit in the audit procedure [26]. However, the short development cycles in DHAs pose a challenge compared to the lengthy periods applicable to established study designs [3]. The benefit assessment and coverage process should, therefore, be planned in such a way as to ensure that the safest possible applications with high quality and proven benefit enter the healthcare system while also providing an incentive for suppliers to invest in developing these applications.

Expert reports on the healthcare system digitisation encourage a wide range of professional and comprehensive training programmes to familiarise GPs with opportunities and conditions involved in integrating DHAs into patient care [22,27,28]. To facilitate healthcare professionals implementing an eHealth intervention, Versluis et al. have provided a practical worksheet to target expected or experienced barriers effectively [23]. Houwink et al. advise incorporating of eHealth education into vocational training and CPD activities [28]. It would also be important for statutory health insurance organisations to advise patients consistently and proactively on using DHAs rather than leaving this to the physicians alone [7, 10, 18].

Various authors explicitly advised expanding on gamification in DHAs: Prioritising motivation, such as by integrating gamification in an intuitive user experience, could make more of a success at initiating lifestyle changes and keeping them in the long term [29,30].

### Strengths and limitations

The study was based on preliminary studies, aligning closely with the GP perspective. The sample comprised GPs organised in specific physicians’ associations and interested in digital technology. Due to the convenience sample, selection bias needs to be considered in all interpretations. This means that attitudes or critical statements of the target group included do not necessarily have to be congruent with GPs not connected to physician networks. In addition, it should be borne in mind that physicians’ associations are prevalent in urban regions, where care structures and conditions for doctors and patients differ from those in rural areas.

Another critical point worth considering is that some participants in the sample were interviewed by telephone rather than a face-to-face interview.

## Conclusion

GPs with experience in health apps rated approved digital health applications (DHAs) favourably regarding healthcare potential and as safer and more reliable than conventional health apps. Many saw benefits to healthcare from using digital health applications. The physicians also saw room for further improvement, especially about usability and gamification as well as extra training programmes and trustworthy information sources for these applications. Interviewees saw the inclusion of DHAs in evidence-based guidelines as a significant step forward.

## Supplementary Material

Supplemental Material

## Data Availability

Research data are available upon request.
