# Supplementary material for: Two years of approved digital health applications in Germany – Perspectives and experiences of general practitioners with an affinity for their use
Source: Eur J Gen Pract. 2023 Mar 15;29(1):2186396. doi: 10.1080/13814788.2023.2186396 (PMC10026738; doi:10.1080/13814788.2023.2186396)
Supplement: Supplemental Material [file IGEN_A_2186396_SM6094.docx]

**Interview guideline**

*I) Introduction*

Physicians have been able to prescribe digital health applications to patients since 2020. These digital health applications aim to contribute towards improvement in diagnosing and treating disease as well as in preventive medicine. These particular applications are considered medical products in contrast to ordinary health apps freely available to patients without prescription. The German Federal Institute for Drugs and Medical Devices (BfArM) audits potential digital health applications and, if they pass the audit, adds them to the DiGA-Verzeichnis, a directory of health applications covered by the German national health system. This requires digital health application manufacturers to submit applications for approval; some criteria need to be met for the application to be included in the directory, which includes standards on data protection and privacy as well as information security, medical information quality, usability, app robustness, and patient safety. The app’s usefulness and added value (effect on healthcare) require adequate documentation.

In our preliminary conversation, you mentioned that you have already used or prescribed digital health applications to patients. In the following, I’d like to ask you a few questions about your current experiences.

*II) Spreading the use of mHealth among patients*

Please give an estimate: What percentage of your patients use health apps and/or other digital tools for healthcare or disease management? Which patient types are involved?

Considering the overall potential of patients, who do you think would be generally interested and willing to use mHealth tools such as conventional health apps, digital health applications and similar software for prevention or disease management. How much of your patient base do you think this would be as a percentage?

How far could you tap into this potential with digital health applications?

*III) Awareness of and attitudes towards digital health applications*

When and how did you first discover that these digital health applications exist?

Were you originally optimistic or sceptical about digital health applications when you found out about them? Why? What, if anything, changed your mind?

As mentioned initially, the German Federal Institute for Drugs and Medical Devices (BfArM) subjects digital health applications to a thorough audit. How far would you expect digital health applications audited by BfArM to be generally reliable software products that doctors would not hesitate to recommend and prescribe to patients?

How far would you expect there to be sufficient legal certainty for physicians prescribing digital health applications and using them in a practical context (regarding potential risk and liability issues)?

How would you rate this: What kind of contribution do you think digital health applications could make in preventive medicine and/or convalescence provided they are used properly, in your opinion or experience?

How much more significant could the contribution that digital health applications make to preventive medicine and/or convalescence be compared to ordinary freely available health apps?

Where do you think digital health applications are (mainly) useful in promising a beneficial effect on patients?

What are the most important benefits of using digital health applications in a clinical setting? Where would you see drawbacks or risks?

I would now like to read out a few statements to you. Please tell me how far you would agree with each statement. Try to rationalise your position as far as possible.

- *‘Digital health applications are too complicated for many patients to use, which could result in false health data being collected and treatment failure in extreme cases’*
- *‘Digital health applications reinforce patient compliance, such as in disease management and prevention’*
- *‘Digital health applications make planning doctor’s appointments more effective’*
- *‘Using digital health applications speeds up the process of identifying and diagnosing diseases and disease risks’*
- *‘Digital health applications would make new patient types more accessible’*

*IV) Prescription practice and experience with use in healthcare*

How did you first come across digital health applications – I mean, how did you decide to use them in patient care? What led you to ‘try out’ digital health applications?

Had you already suggested to patients that they use conventional health apps before you started on digital health applications? How far have you increased prescriptions since the existence of digital health applications? Have you gained more confidence and safety from digital health applications?

How often would you say you have raised the possibility of support using digital health applications in preventive medicine or disease management in patient consultations?

How often would you say you have recommended certain digital health applications for prevention and/or treatment in patients and then actually prescribed them?

Where has digital health application regulation mainly taken place, in what application areas and scenarios? (Examples include prevention, lifestyle changes, disease management)

How much do you use digital health applications specifically for treatment planning, that is, relying on data collected from a health app for inclusion in treatment?

Casting your mind back, can you remember prescribing certain digital health applications more than others to patients?

Which patient types are especially receptive to digital health applications and which are not?

What criteria would you say a digital health application needs to satisfy for you to recommend or prescribe it to your patients? What criteria do you see as especially important? Please name one to five criteria.

Thinking of application areas where you’ve used digital health applications: How do you rate their benefit? Where was the benefit most significant?

What health effects have you already seen from patients using digital health applications? What kinds of adverse effects have you seen, if any?

*V) Information behaviour and personal competence assessment*

How often do you look for information on digital health applications, conventional health apps and/or other digital tools?

What sources (such as websites) do you use to gain information?

How would you rate your knowledge and ability to gaugeg the range of digital health applications available?

How would you rate your knowledge and ability to advise your patients on applications?

*VI) Assessment, further development, and prevalence of digital health applications*

Judging from your previous experience with digital health applications, how do you think digital health applications could be improved, and what would you especially like to see?

Assuming the points you indicated were implemented, would you be willing to consider digital health applications more often than before in patient care if these changes were to be implemented?

How far would you expect digital health applications to benefit healthcare and medical practices in a beneficial and meaningful way in the medium and long term? How far would you say this is a realistic expectation?

There have been signs that many registered physicians and general practitioners in particular have been reluctant in their approach towards digital health applications, that is, they’ve not yet been prescribing them. Where do you think the reasons for this lie?

How do you think we could go about changing this?
